# Supplementary material for: Ultra-permeable silk-based polymeric membranes for vacuum-driven nanofiltration
Source: Nat Commun. 2024 Oct 5;15:8656. doi: 10.1038/s41467-024-53042-6 (PMC11455960; doi:10.1038/s41467-024-53042-6)
Supplement: Supplementary file 1 — Supplementary Information [file 41467_2024_53042_MOESM1_ESM.pdf]

Supplementary Information

Ultra-permeable Silk-based Polymeric Membranes for Vacuum-driven Nanofiltration

Bowen Gan[1], Lu Elfa Peng[1], Wenyu Liu[1], Lingyue Zhang[1], Li Ares Wang[1], Li Long[1], Hao Guo[2], Xiaoxiao Song[3], Zhe Yang[1,4]✉, Chuyang Y. Tang[1]✉

[1]Department of Civil Engineering, The University of Hong Kong, Pokfulam, Hong Kong SAR, China.
[2]Institute of Environment and Ecology, Shenzhen International Graduate School, Tsinghua University, Shenzhen, China.
[3]Centre for Membrane and Water Science and Technology, Ocean College, Zhejiang University of Technology, Hangzhou, China.
[4]Dow Centre for Sustainable Engineering Innovation, School of Chemical Engineering, The University of Queensland, Brisbane, QLD 4072, Australia.

✉ Corresponding Author:
zheyang@connect.hku.hk (Z. Yang)
tangc@hku.hk (C. Tang)

Table of Contents

Supplementary Information 1

1. Supplementary Methods 1

2. Supplementary Figures 2

Supplementary Fig. 1 The fabrication process of substrate-templated SNF-NF membranes. 2

Supplementary Fig. 2 The fabrication process of SNF. 3

Supplementary Fig. 3 The characterization of SNF.. 4

Supplementary Fig. 4 SEM images of the surface morphology of substrates with different treatment.. 5

Supplementary Fig. 5 The FTIR results of substrates with different treatments 6

Supplementary Fig. 6 Morphologies of the pristine and the SNF-coated PVDF substrates with different mass loading. 7

Supplementary Fig. 7 SEM images for the surface of SNF0-NF0.5 membranes with different resolution 8

Supplementary Fig. 8 The pores distribution of PVDF MF substrate. 9

Supplementary Fig. 9 Cross-sectional SEM micrographs of the control PVDF, the SNF-coated PVDF substrates with different mass loading 10

Supplementary Fig. 10 Surface SEM images of substrates 11

Supplementary Fig. 11 The contact angle results of pristine and SNF-coated PSF substates 11

Supplementary Fig. 12 The dynamic water contact angle of the pristine PVDF, SNF20-PVDF substrates, and SNF20-NF0.5 membranes 12

Supplementary Fig. 13 STEM−EDX elemental mappings of the control SNF0-NF0.5 membrane. 13

Supplementary Fig. 14 Cross-sectional SEM micrographs of the control PVDF, SNF20-PVDF substrates to SNF20-NF0.5 membranes 14

Supplementary Fig. 15 Morphology changes of the substrate-templated NF membranes with different SNF coating mass formed by IP reaction of 0.5 wt% PIP with 0.1 wt% TMC. 15

Supplementary Fig. 16 The Doppler broadening energy spectroscopy results of SNF0-NF0.5 and SNF20-NF0.5. 16

Supplementary Fig. 17 Bright-field TEM images of the cross-section of SNF0-NF0.5 and SNF20-NF0.5. 17

Supplementary Fig. 18 Comparison of the interaction energy and the average number of hydrogen bonds between PIP-PVDF and PIP-SNF. 18

Supplementary Fig. 19 Bright-field TEM images of the cross-section of SNF0-NF1. 19

Supplementary Fig. 20 Quartz crystal microbalance with dissipation (QCM-D) release test of PIP monomer for bare and SNF-coated sensor.. 20

Supplementary Fig. 21 The influence of SNF loading mass and IP reaction time on the membrane separation performance based on the SNF20-PVDF substrates. 21

Supplementary Fig. 22 The impact of coating mass on SNF substrates. 22

Supplementary Fig. 23 Bright-field TEM images of the cross-section of SNF20-NF0.5, SNF20-NF0.2* and SNF20-NF0.1*.. 23

Supplementary Fig. 24 The separation performance of SNF0-NF0.1* and SNF0-NF0.2*. 24

Supplementary Fig. 25 The surface morphology of SNF20-NF0.1* membranes.. 25

Supplementary Fig. 26 The surface morphology for the PES substrate and NF membrane based on the SNF-coated PES substrate. 26

Supplementary Fig. 27 The water permeance of PVDF MF and PES MF substrates. 27

Supplementary Fig. 28 Separation performance of PES-NF membranes with and without SNF.. 28

Supplementary Fig. 29 The MWCO characterization for SNF20-NF0.1* membranes. 29

Supplementary Fig. 30 Separation performance of NF270 membranes for five different salts and $CaCl_2/Na_2SO_4$ selectivity under the submerged vacuum-driven NF mode.................................. 30

Supplementary Fig. 31 The PFASs rejection for SNF20-NF0.1* membranes 31

Supplementary Fig. 32 Digital photo a vacuum filtration apparatus. 32

Supplementary Fig. 33 Digital photo of membrane cell... 33

Supplementary Fig. 34 Separation performance of NF270 membranes for five different salts under the cross-flow filtration mode.. 34

Supplementary Fig. 35 Separation performance of SNF20-NF0.1* membranes for five different salts under the cross-flow filtration mode. 34

Supplementary Fig. 36 The separation performance of SNF20-NF0.1* under different operating conditions. 35

Supplementary Fig. 37 The influence of operational pressure on the membrane separation performance of SNF20-NF0.1* in two processes.. 36

Supplementary Fig. 38 The influence of the feed concentration of Na_2SO_4 on the membrane separation performance of SNF20-NF0.1*... 37

Supplementary Fig. 39 The The long-term separation performance of SNF20-NF0.1*.. 37

3. Supplementary Tables.. 39

Supplementary Table 1. Elemental compositions of the top surface of PVDF and SNF-PVDF substrates measured by XPS.. 39

Supplementary Table 2. The fabrication recipe for substrates with and without SNF.................. 39

Supplementary Table 3. The fabrication recipe for SNF-NF membranes with and without SNF. . 39

Supplementary Table 4. Operating parameters assumed in the calculation of SEC. 40

Supplementary Table 5. A comparison of S parameters of DBES between this study and other nanofiltration studies in the literature... 41

Supplementary Table 6. A comparison of MWCO between this study and other polyamide TFC NF membranes reported in the literature.. 42

Supplementary Table 7. The Na_2SO_4 and $MgSO_4$ rejection of polyamide NF membranes. 43

4. Supplementary References 45

1. Supplementary Methods

Chemicals and Materials

Trimesoyl chloride (TMC, 98%), piperazine (PIP, 99%), and n-hexane were purchased from Sigma-Aldrich. Sulfate anhydrous (Na_2SO_4), sodium chloride (NaCl), magnesium chloride hexahydrate ($MgCl_2 \cdot 6H_2O$), Calcium chloride ($CaCl_2$), and magnesium sulfate ($MgSO_4$) were purchased from Dieckmann (Hong Kong, Chemical Industry co., Ltd.). Polyfluoroalkyl substances (PFASs) analyzed in this study include perfluorobutyric acid (PFBA, Alfa Aesar), potassium nonafluoro-1-butanesulfonate (PFBS potassium salt, TCI), perfluoro (2-methyl-3-oxahexanoic) acid (Genx, Macklin), sodium perfluorooctanoate (PFOA sodium salt, Alfa Aesar), and potassium perfluorooctanesulfonate (PFOS potassium salt, Sigma-Aldrich). Deionized (DI) water is produced by the Millipore's purification system for use. Hydrophilic PVDF MF membranes (0.22 μm) were also obtained from Millipore. Commercial NF membranes (NF270) were purchased from DuPont FilmTech.

2. Supplementary Figures

The fabrication process of substrate-templated NF-SNF membranes mainly involves the fabrication of SNF-coated substrates by the spraying-coated method and the construction of a PA layer by the IP method (Supplementary Fig. 1).

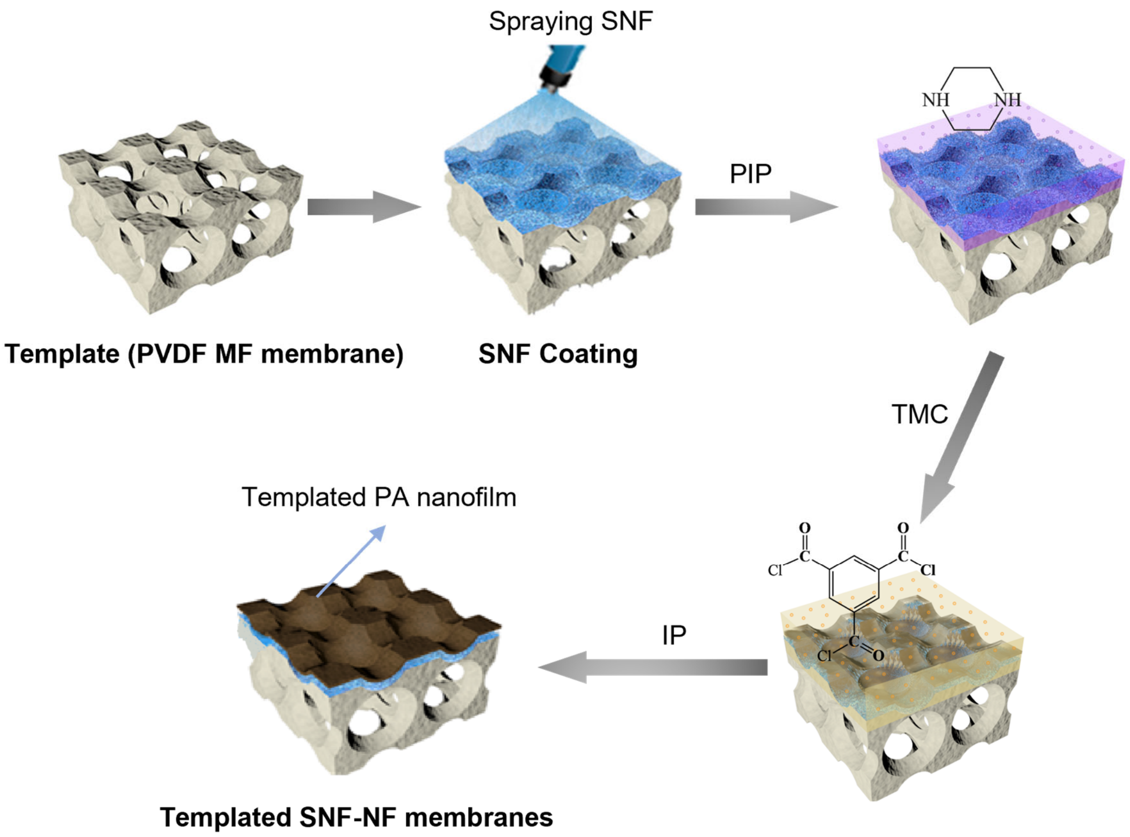

Supplementary Fig. 1 | The fabrication process of substrate-templated SNF-NF membranes. The SNF water suspension was first coated on a PVDF MF substrate with care. Specifically, the back side of substrate was first adhered on a multifunctional porous plate at the heating temperature of 45°C with the aid of a suction force to avoid the accumulation SNF suspension on the substrate surface. To obtain the resultant NF membrane, IP reaction was performed between PIP aqueous solution and TMC organic solution

Supplementary Fig. 2 briefly shows the fabrication process of SNF from the silkworm fibers to the SNF suspension. The raw silk contains surface sericin covering the core fibroin. When the surface sericin protein was removed, the fibroin can be further processed into nano-size silk nanofibers (SNF).

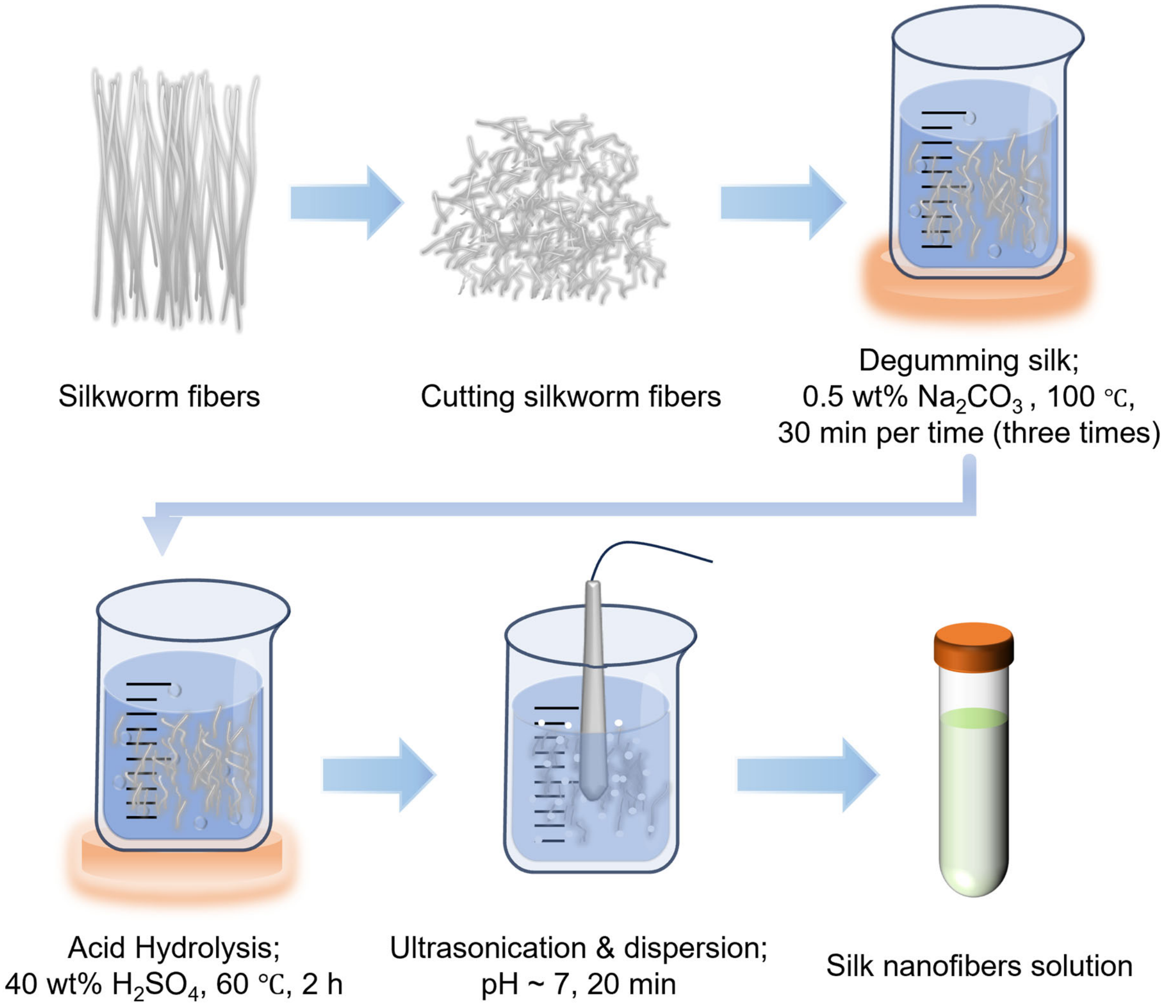

Supplementary Fig. 2 | The fabrication process of SNF.

The resulting SNF suspension showed the Tyndall effect (Supplementary Fig. 3A), indicating excellent dispersibility in water. The dimension of nano-sized SNFs was 9.6 ± 2.3 nm in width and 380 ± 200 nm in length measured by NanoScope Analysis software (Supplementary Fig. 3B-E). Furthermore, the fourier transform infrared spectroscopy (FTIR) spectra results of SNF film and degummed silk fibers (Supplementary Fig. 3F) both demonstrate β-sheets (around 1514, 1618, and 1647 cm^{-1}) and β-turns (around 1695 cm^{-1})[1] molecular structures.

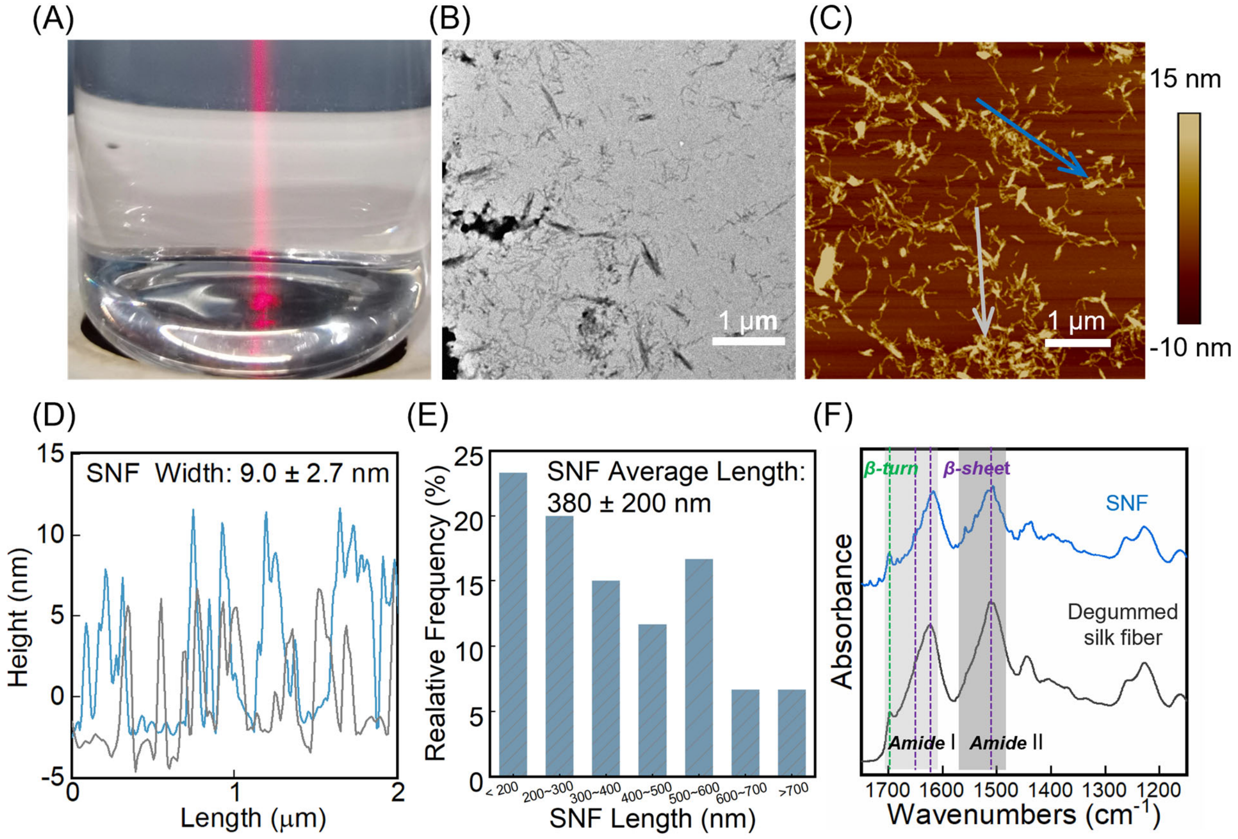

Supplementary Fig. 3 | The characterization of SNF. (**A**) Tyndall effect in the SNF suspension, (**B**) the TEM image of SNF, (**C**) The AFM image of SNF, (**D**, **E**) their corresponding statics results of SNF dimensions, and (F) the FTIR results of SNF film and degummed silk fibers. The error bars of SNF width and average length represent the standard deviation of the test data from distinct samples (n = 12 and n = 60, respectively).

We have designed experiments to examine the stability of the SNF coating layer. Specifically, an SNF-coated PVDF substrate was immersed in TMC-hexane solution for 1 minute and then vigorously stirred in DI water for 10 minutes. This immersion-stirring process was repeated for three times. For comparison, another SNF-coated PVDF substrate was directly stirred in DI water without treatment with a TMC-hexane solution. We further compared the surface morphology change of these substrates by SEM characterization (Supplementary Fig. 4). The SEM characterization revealed that the SNF coating treated with TMC solution remained complete on the substrate after washing, whereas the control SNF coating was washed away. FTIR results (Supplementary Fig. 5) also validated that the characteristic SNF peak persisted on TMC-treated coating layers following water washing, demonstrating the excellent stability of SNF coating layers. This improved stability may be caused by the crosslinking of SNF by TMC through the reaction of the abundant amine groups of SNF with the acyl chloride groups of TMC

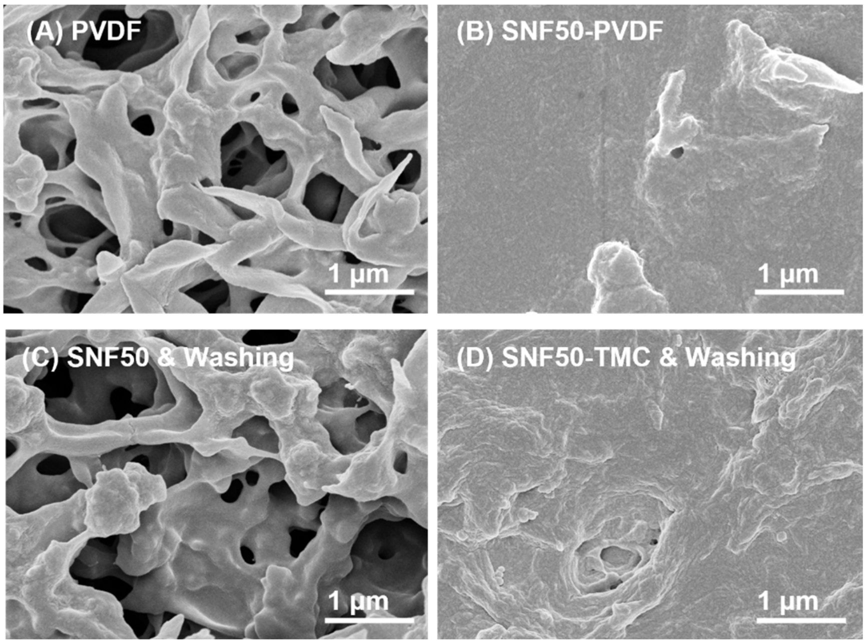

Supplementary Fig. 4 | SEM images of the surface morphology of substrates with different treatments. (**A**) Pristine PVDF substrate (**B**) SNF-coated substrate (**C**) SNF-coated substrate washed by DI water (**D**) SNF-coated substrate treated with TMC solution, then washed by DI water.

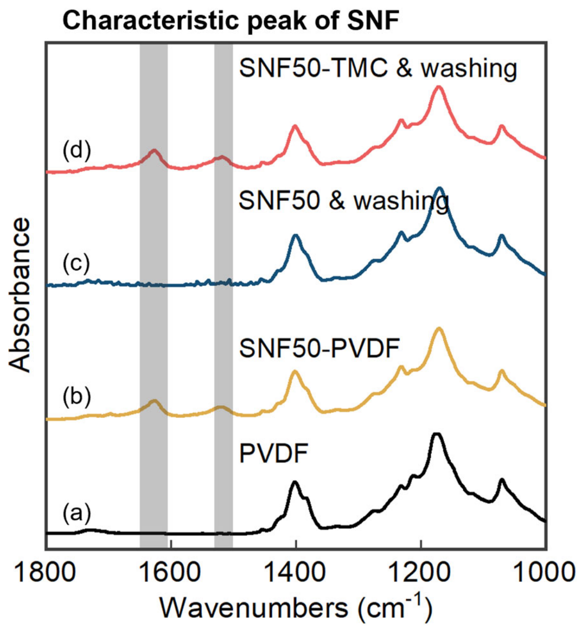

Supplementary Fig. 5 | The FTIR results of substrates with different treatments: (a) The pristine PVDF substrate; (b) SNF-coated substrate; (c) SNF-coated substrate washed by DI water; and (d) The SNF-coated substrate treated with TMC solution, then washed by DI water.

Supplementary Fig. 6 presented the influence of varying SNF loading mass on the substrate morphology. With the additional increase of SNF loading mass from 0, 41, 61 to 102 μg cm^{-2}, the micropores of the pristine substrates were gradually diminished, enabling the substrate surface to become smoother.

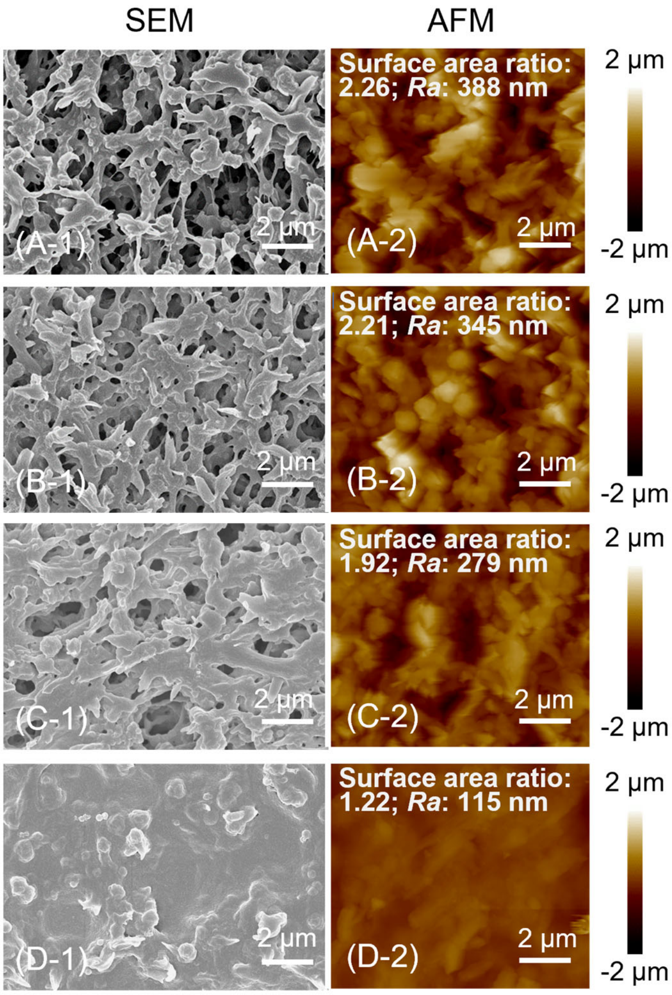

Supplementary Fig. 6 | Morphologies of the pristine and the SNF-coated PVDF substrates with different mass loading: 0 μg cm^{-2} (**A-1, A-2**), 41 μg cm^{-2} (**B-1, B-2**), 61 μg cm^{-2} (**C-1, C-2**) to 102 μg cm^{-2} (**D-1, D-2**).

Supplementary Fig. 7 showed that defects appeared on the surface of SNF0-NF0.5 membranes (control NF membranes) in different resolutions. It is worthwhile to note that SEM sample preparation involves sputter coating by Au and Pt of approximately 5 nm [2], which may seal some small defective regions. Furthermore, the limited resolution of SEM means small defects of a few nanometers are difficult to be detected. Therefore, the actual proportion of defective regions in the PA could be larger than that of observed from the SEM images.

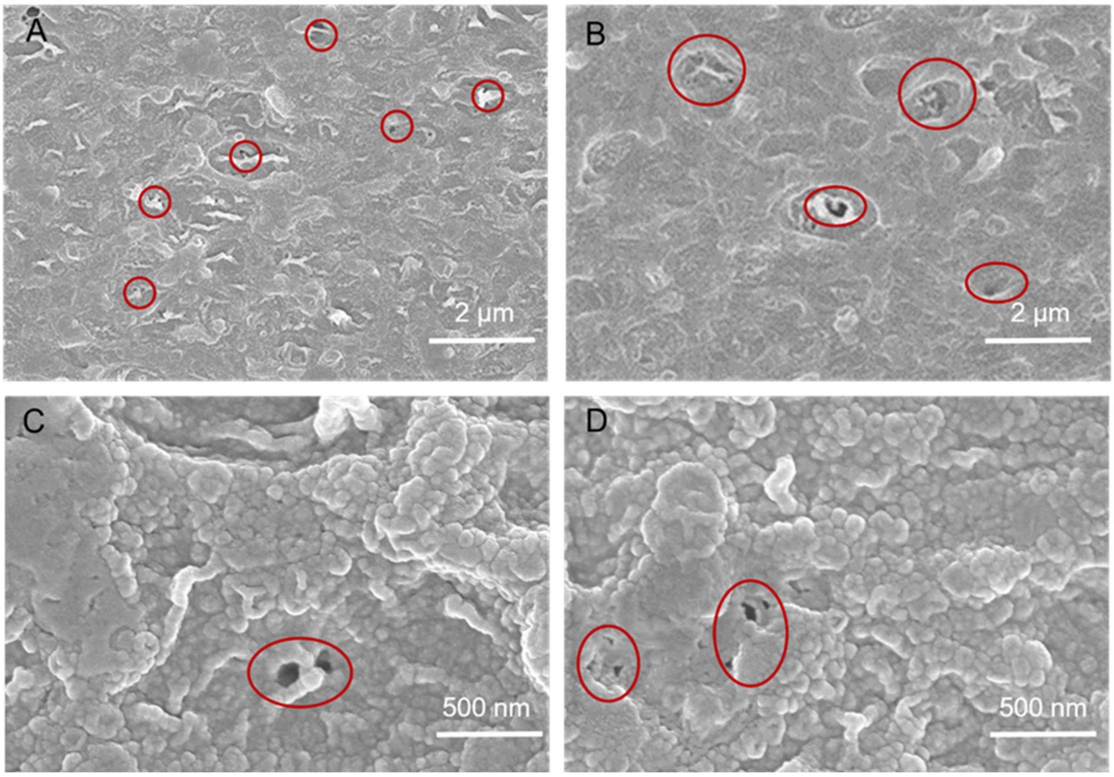

Supplementary Fig. 7 | SEM images for the surface of SNF0-NF0.5 membranes with different resolution. The Scale bar for (**A-B**) is 2 μm, while the scale bar for (**C-D**) is 500 nm. Defects are marked by circles in these micrographs.

Supplementary Fig. 8 exhibited the pores distribution of PVDF MF substrate.

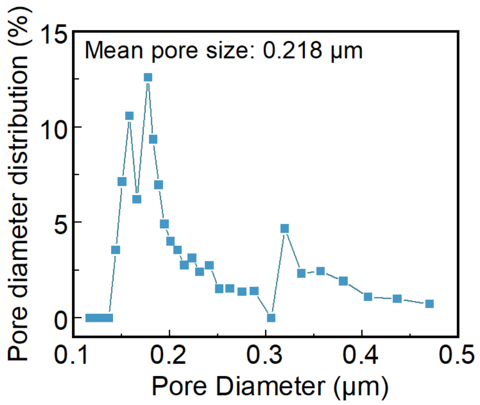

Supplementary Fig. 8 | The pores distribution of PVDF MF substrate. The pore size distribution was measured using a Pore Size Analyzer (BSD-660S, BSD Instrument Co., Ltd).

Supplementary Fig. 9 exhibited the thickness of SNF coting with different loading mass.

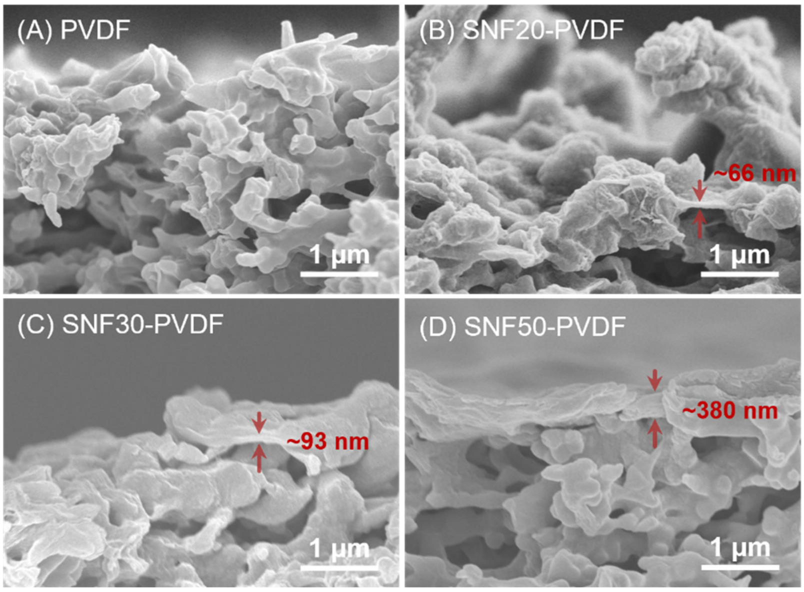

Supplementary Fig. 9 | Cross-sectional SEM micrographs of the control PVDF, the SNF-coated PVDF substrates with different mass loading: 0 μg cm^{-2} (**A**)**,** 41 μg cm^{-2} (**B**)**,** 61 μg cm^{-2}, (**C**) to 102 μg cm^{-2} (**D**).

Supplementary Fig. 12 shows the dynamic contact angle results of PVDF and SNF20-PVDF. For both substrates, the contact angle approached zero at time longer than approximately 10s, which is likely to due to the highly porous surface of the two substrates. Nevertheless, the SNF20-PVDF had a faster decrease in the contact angle values. To avoid interference from the highly porous surface of the substrates, we coated SNF on a polysulfone (PSF) UF membranes with small surface pore size and a low surface porosity (Supplementary Fig. 10) for water contact angle measurement (time = 10s). As shown in Supplementary Fig. 11, compared with the pristine PSF, SNF-coated PSF substrates exhibited a significantly improved surface hydrophilicity with a lower contact angle of 60.7°, compared to the pristine PSF substrate with a contact angle of 80.2°. This observation suggested the SNF coating imparted increased hydrophilicity to the substrate surface.

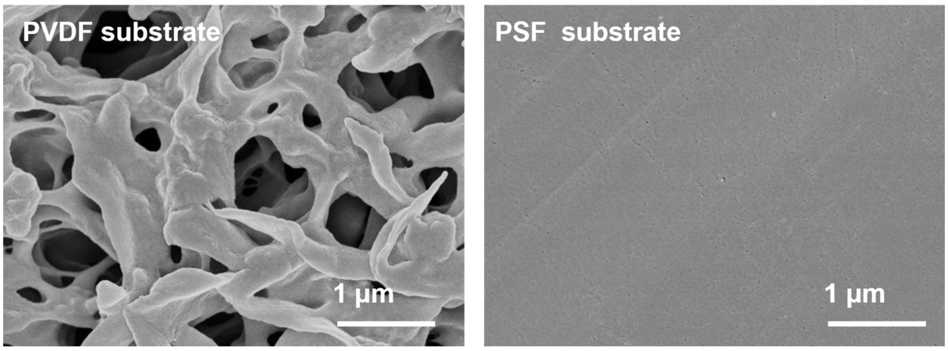

Supplementary Fig. 10 | Surface SEM images of PVDF and PSF substrates

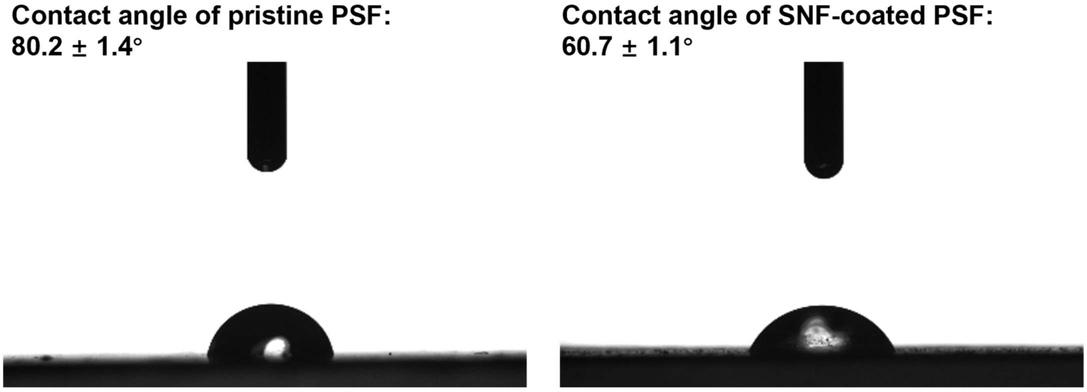

Supplementary Fig. 11 | The contact angle results of pristine and SNF-coated PSF substates.

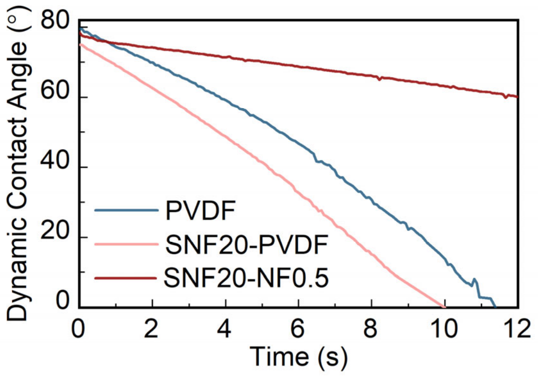

Supplementary Fig. 12 | The dynamic water contact angle of the pristine PVDF, SNF20-PVDF substrates, and SNF20-NF0.5 membranes.

The TEM-EDX characterization of the cross-section of SNF0-NF0.5 demonstrated the presence of only a PA layer spanning over the substrate pore (Supplementary Fig. 13).

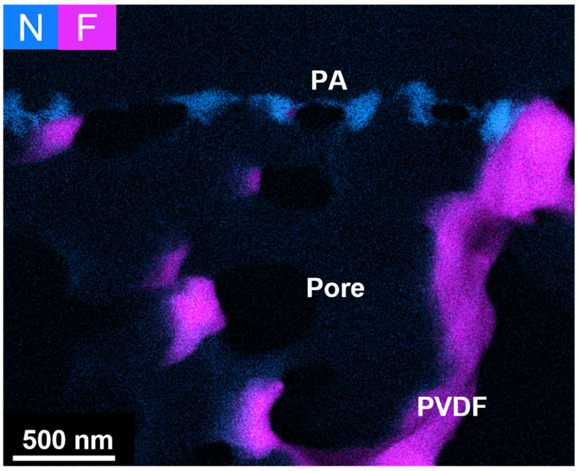

Supplementary Fig. 13 | STEM−EDX elemental mappings of the control SNF0-NF0.5 membrane. "N" (bright blue) denotes nitrogen and "F" (pink) denotes fluorine.

We examined the cross-sectional morphologies of the substrates (with and without SNF) and the SNF-NF0.5 membrane by SEM in Supplementary Fig. 14. Compared with pristine PVDF substrates, numerous downy structures appeared on the surface of SNF20-PVDF, corroborating the formation of the SNF coating layer. After the IP reaction (SNF20-NF0.5), the top surface of SNF20-PVDF covered a continuous nanofilm with a thickness of approximately 23 nm, which is in good agreement with the measured results in TEM images (Fig. 1E-2).

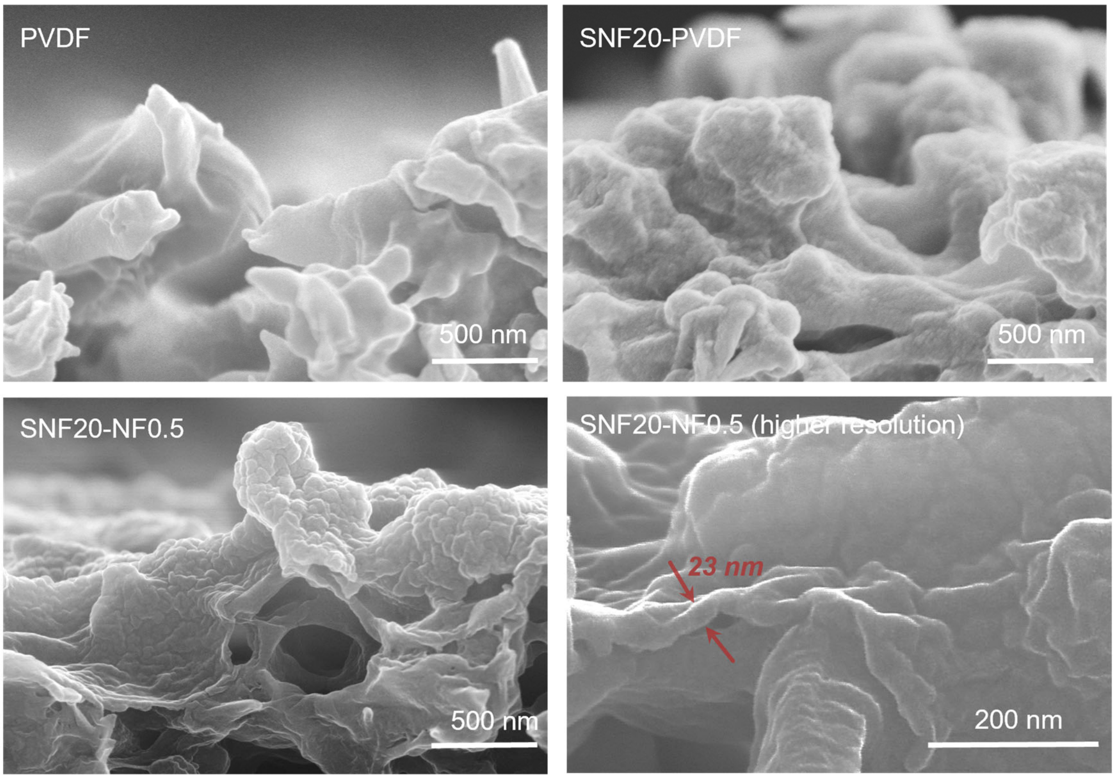

Supplementary Fig. 14 | Cross-sectional SEM micrographs of the control PVDF, SNF20-PVDF substrates to SNF20-NF0.5 membranes.

As shown in Supplementary Fig. 15, the surface area ratio and roughness (*Ra*) of SNF-NF decreased with the additional SNF, which exhibited a similar tendency in corresponding SNF-coated substrates.

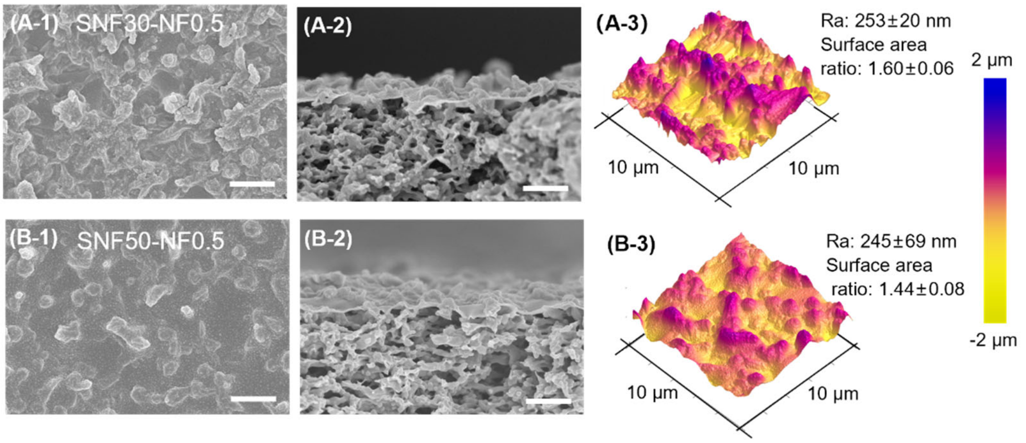

Supplementary Fig. 15 | Morphology changes of the substrate-templated NF membranes with different SNF coating mass formed by IP reaction of 0.5 wt% PIP with 0.1 wt% TMC. Surface (**A-1**) and cross-section (**A-2**) SEM image and AFM results (**A-3**) of SNF30-NF0.5. Surface (**B-1**) and cross-section (**B-2**) SEM images and AFM results (**B-3**) of SNF50-NF0.5.

Supplementary Fig. 16 showed Doppler broadening energy spectroscopy (The Institute of High Energy Physics, Beijing, China) of SNF0-NF0.5 (without SNF) and SNF20-NF0.5 (with SNF) membranes.

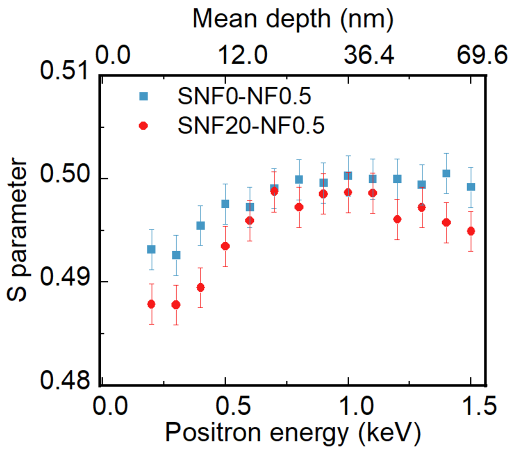

Supplementary Fig. 16 | The Doppler broadening energy spectroscopy results of SNF0-NF0.5 and SNF20-NF0.5.

Supplementary Fig. 17 exhibited the thickness of PA in SNF0-NF0.5 and SNF20-NF0.5 membranes measured by TEM characterization.

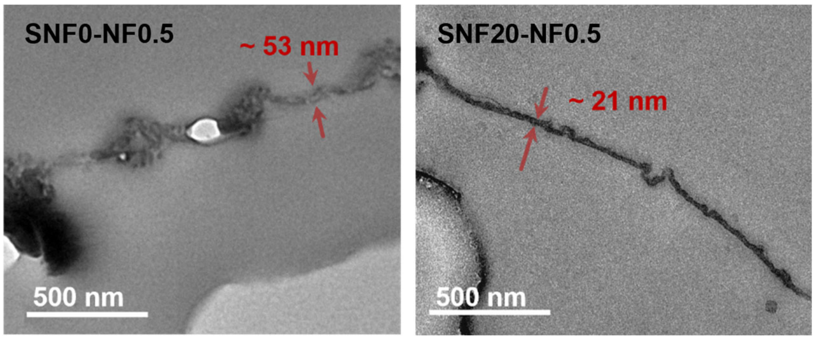

Supplementary Fig. 17 | Bright-field TEM images of the cross-section of SNF0-NF0.5 and SNF20-NF0.5.

We also compared the interaction energy change over the 50 ns simulation time between the PIP-PVDF system and the PIP-SNF system (Supplementary Fig. 18A and 18 B). Generally, the PIP molecules can be absorbed by the SNF fraction more strongly than the PVDF fraction. Meanwhile, the number of hydrogen bonds formed in the PIP-SNF system is significantly higher than that in the PIP-PVDF system (Supplementary Fig. 18C).

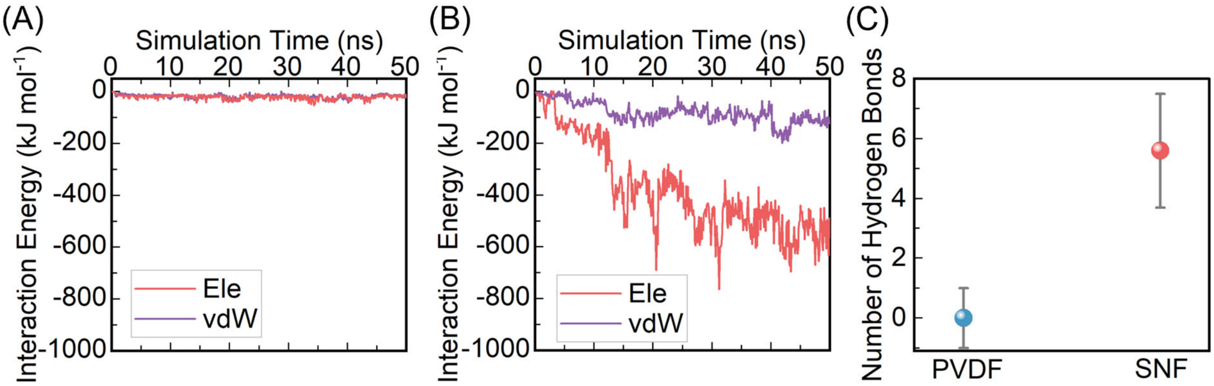

Supplementary Fig. 18 | Comparison of the interaction energy and the average number of hydrogen bonds between PIP-PVDF and PIP-SNF: the details of the interaction energy including electrostatic (Ele) and van der Waals (vdW) forces in PVDF-PIP (**A**) and SNF-PIP (**B**) systems, respectively. (**C**) the simulated data of the average number of hydrogen bonds counted by the last 10 nanoseconds (ns).

We have prepared the NF membranes with a higher PIP concentration of 1 wt% and 0.1 wt% TMC on the pristine PVDF support (SNF0-NF1). The PA layer was approximately 75 nm in thickness (Supplementary Fig. 19), which was much greater compared to those for SNF0-NF0.5 (53 nm) and SNF20-NF0.5 (21 nm). For the membrane SNF20-NF0.5, even though the SNF layer greatly increased the absorption of PIP, its PA thickness remained relatively thin. This observation confirms the advantages of the SNF layer for creating ultrathin PA films.

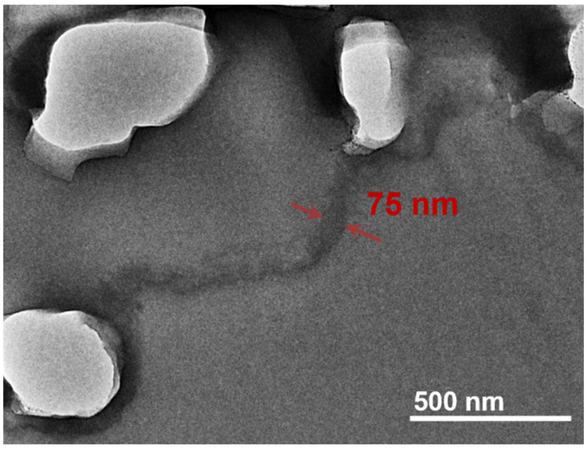

Supplementary Fig. 19 | Bright-field TEM images of the cross-section of SNF0-NF1. (IP condition: 1 wt% of TMC and 0.5 wt% of PIP; reaction time of the 60 s.)

We further performed QCM-D tests to investigate the influence of SNF on the desorption behavior of PIP monomers. Specifically, a quartz crystal sensor with or without SNF coating was mounted into the QCM-D chambers. In order to achieve an identical initial loading of PIP (approximately 1500 ng cm^{-2}), we used a 8 wt % PIP solution for the control sensor without SNF and a 0.5 wt % PIP solution for the SNF-coated sensor. After stabilization, DI water was introduced into the chambers to determine the kinetics of PIP desorption. As shown in Supplementary Fig. 20, the SNF coating could significantly reduce the released rate of PIP.

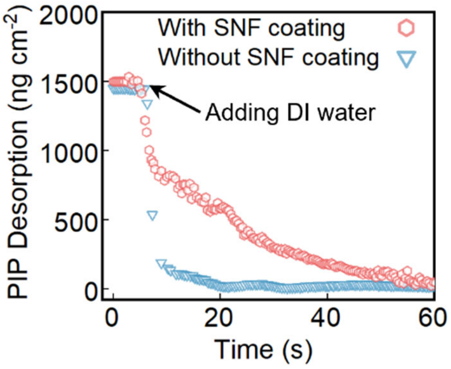

Supplementary Fig. 20 | Quartz crystal microbalance with dissipation (QCM-D) release test of PIP monomer for bare and SNF-coated sensor.

We investigated the influence of SNF loading mass on the membrane separation performance (Supplementary Fig. 21A). When the SNF loading mass increased from 41, 61 to 102 μg cm^{-2}, the water permeance of SNF-NF membranes significantly decreased with a slight change in Na_2SO_4 rejection. The influence of IP reaction time on the membrane separation performance was investigated, with IP reaction time of 30 seconds showing the best separation performance (Supplementary Fig. 21B)

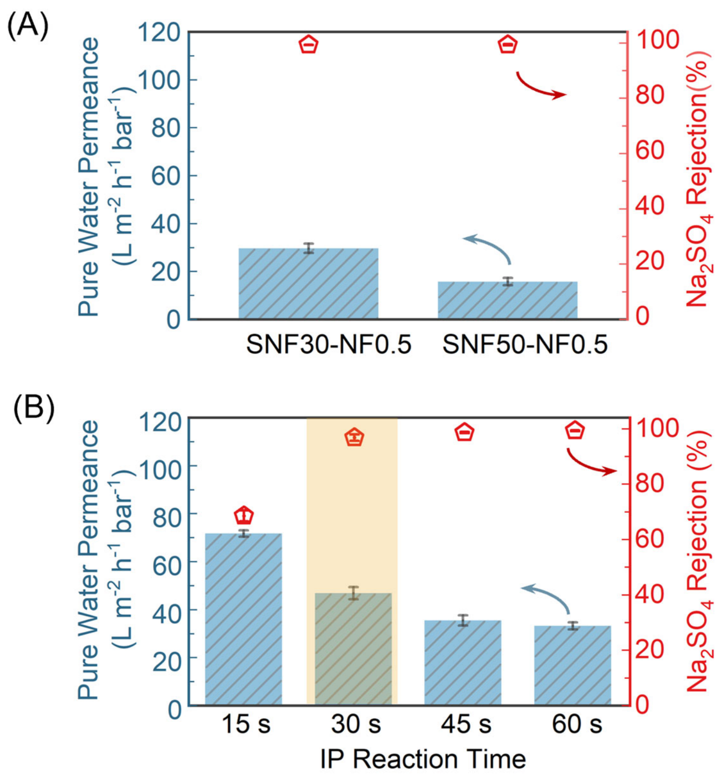

Supplementary Fig. 21| The influence of SNF loading mass and IP reaction time on the membrane separation performance based on the SNF20-PVDF substrates. (**A**) Pure water permeance and Na_2SO_4 rejection with the additional increased SNF loading mass at 0.1 wt% TMC and 0.5 wt% PIP with 60s IP reaction time. (**B**) Pure water permeance and Na_2SO_4 rejection with the increased IP reaction time at 0.1 wt% TMC and 0.5 wt% PIP. The rejection test was performed using a feed solution of 1000 ppm Na_2SO_4 and pure water permeance was determined using DI water. The yellow shading represented the optimized reaction time. The filtration test adopted a conventional pressure-driven cross-flow mode with an applied hydraulic pressure of 3 bar. The error bars of separation performance represent the standard deviation of the test date from three distinct samples (*n* = 3).

We investigated the impact of SNF loading mass on PVDF substrates. Supplementary Fig. 22 demonstrates that the excessive loading mass of SNF results in a sharp decrease in the water permeance of the substrate.

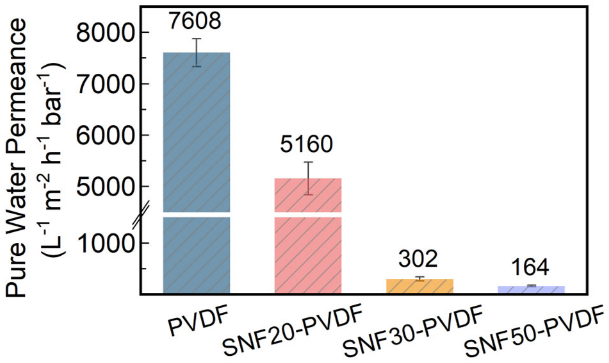

Supplementary Fig. 22 | The impact of coating mass on SNF substrates. The water permeance of substrates with a different coating mass of SNF; The filtration test adopted a dead-end mode with an applied hydraulic pressure of 0.5 bar. The error bars of water permeance represent the standard deviation of the test date from three distinct samples (*n* = 3).

The PA thickness in SNF20-NF0.2* and SNF20-NF0.1* have been measured based on TEM cross-section images. As shown in Supplementary Fig. 23, after reducing the IP reaction and PIP concentration, the PA thickness of SNF20-NF0.1* was reduced to approximately 14 nm.

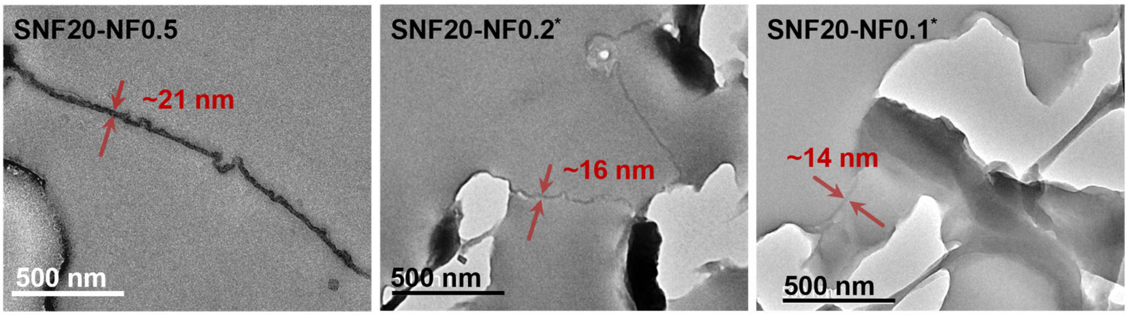

Supplementary Fig. 23 | Bright-field TEM images of the cross-section of SNF20-NF0.5, SNF20-NF0.2*, and SNF20-NF0.1*.

As shown in Supplementary Fig. 24, both SNF0-NF membranes without SNF coating exhibited much lower Na_2SO_4 rejection (<40%) compared to the corresponding SNF-incorporated SNF-NF membranes (Fig. 3A), confirming that the SNF coatings played a dominant role in improving rejection.

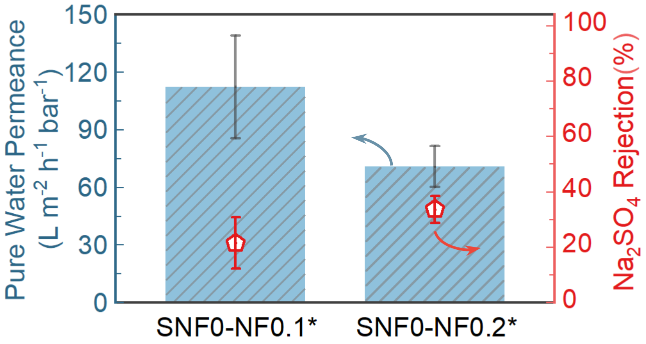

Supplementary Fig. 24 | The separation performance of SNF0-NF0.1* and SNF0-NF0.2*. Rejection test condition: 1000 ppm Na_2SO_4, applied pressure of 3 bar. The error bars of water permeance represent the standard deviation of the test date from three distinct samples.

After optimizing the IP condition, the SNF20-NF0.1* membranes still maintain a high membrane roughness (*R*a = 299 ± 35) and surface area ratio (1.57 ± 0.05), which enlarges the membrane filtration area and could contribute to the enhancement of water permeance (Supplementary Fig. 25).

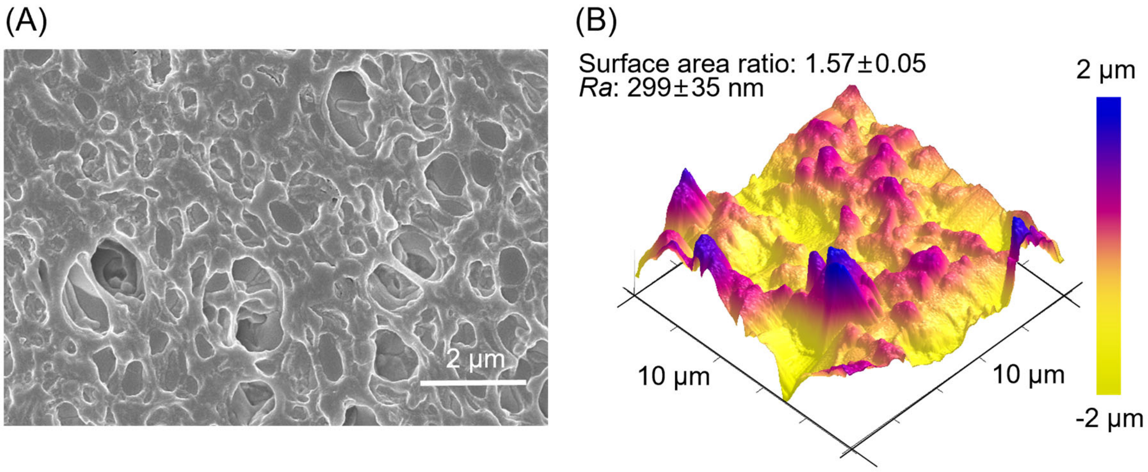

Supplementary Fig. 25 | The surface morphology of SNF20-NF0.1* membranes. (**A**) membrane surface SEM image and (**B**) AFM image. The error bars represent the standard deviation of data from three distinct samples (*n* = 3).

Supplementary Fig. 26 showed the morphology of PES substrate and NF membrane.

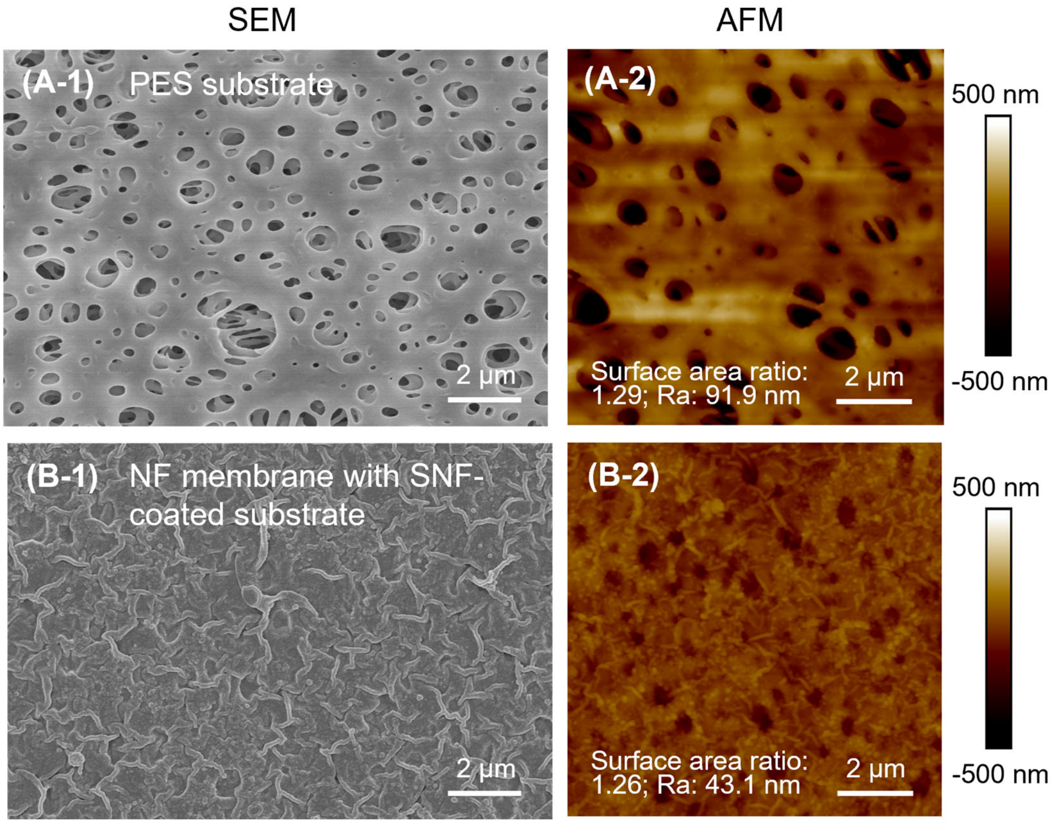

Supplementary Fig. 26 | The surface morphology for the PES substrate and NF membrane based on the SNF-coated PES substrate. Surface SEM images (**A-1**) and AFM results (**A-2**) of PES substrate. Surface SEM images (**B-1**) and AFM results (**B-2**) of NF membrane with SNF-coated PES substrates. (SNF loading mass: 41 μg cm^{-2}; IP condition: 0.5 wt% of PIP and 0.1 wt% of TMC, reaction time: 60 s)

Supplementary Fig. 27 demonstrated water permeance of PES MF (19360 ± 1550 L m^{-2} h^{-1} bar^{-1}) is much higher than that of PVDF MF substrates (7680 ± 271 L m^{-2} h^{-1} bar^{-1}).

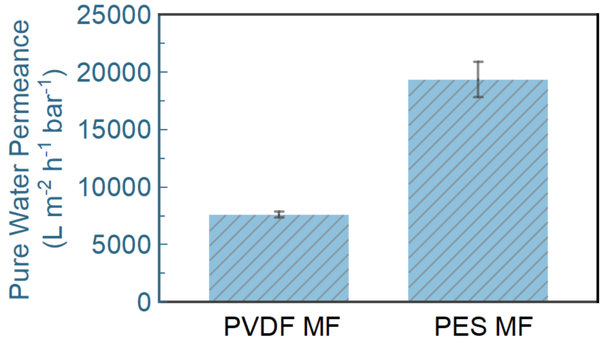

Supplementary Fig. 27 | The water permeance of PVDF MF and PES MF substrates. The filtration test adopted a dead-end mode with an applied hydraulic pressure of 0.5 bar. The error bars of water permeance represent the standard deviation of the test date from three distinct samples (*n* = 3).

Supplementary Fig. 28 shows the separation performance of PES-NF membranes with/without SNF. The PES-NF membranes with SNF demonstrated a marginal increase in water permeance compared to the control counterpart.

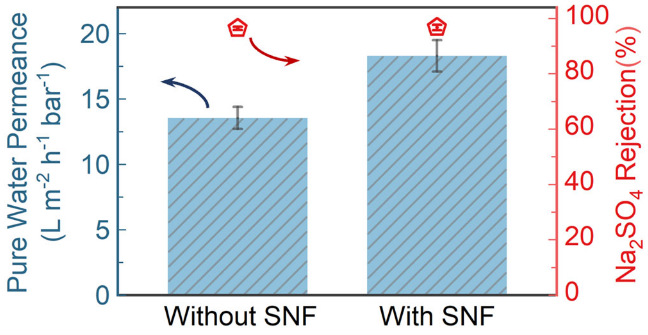

Supplementary Fig. 28 | Separation performance of PES-NF membranes with and without SNF. PES-NF membranes were formed by IP reaction of 0.5 wt% PIP with 0.1 wt% TMC on the PES MF substrates with/without an SNF coating. The rejection test was performed using a feed solution of 1000 ppm Na_2SO_4 and pure water permeance was determined using DI water. The filtration test adopted a conventional pressure-driven cross-flow mode with an applied hydraulic pressure of 3 bar. The error bars represent the standard deviation of the rejection rate or water permeance from the three distinct samples (*n* = 3).

MWCO has been widely used to characterize the pore size property of the PA NF membranes, and MOCO is related to the cross-linking or density of PA. Supplementary Fig. 29 indicated that the MWCO of SNF20-NF0.1* membranes was 633 Da, which is comparable to many PA TFC NF membranes reported in the literature (Supplementary Table 5)

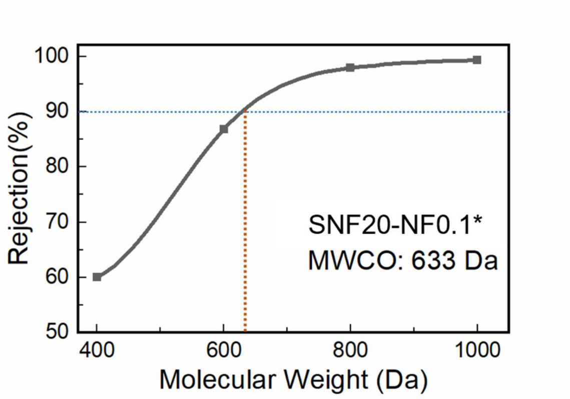

Supplementary Fig. 29 | The MWCO characterization for SNF20-NF0.1* membranes. The presented data is the averaged value based on three distinct samples (*n* = 3).

Under the submerged vacuum-driven NF mode, NF270 demonstrated a high Na_2SO4 rejection (> 96%) and moderate rejections (< 30%) for $MgCl_2$ and $CaCl_2$ (Supplementary Fig. 30), which results in low $CaCl_2/Na_2SO_4$ selectivity compared to our SNF20-NF0.1* membranes.

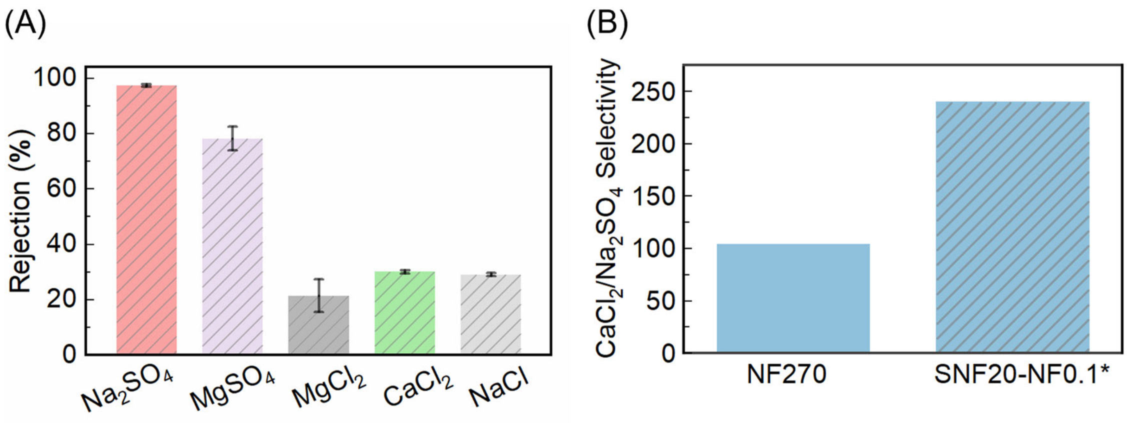

Supplementary Fig. 30 | Separation performance of NF270 membranes for five different salts and $CaCl_2/Na_2SO_4$ selectivity under the submerged vacuum-driven NF mode. (**A**) The rejection of NF270 membranes for five different salts. (**B**) $CaCl_2/Na_2SO_4$ selectivity of NF270 and SNF20-NF0.1* membrane. The error bars represent the standard deviation of the salt rejection rate from the measurement data of three distinct samples (*n* = 3, testing condition: single salt concentration of 500 ppm at a vacuum pressure of 0.9 bar).

The separation performance of SNF20-NF0.1* membranes in the submerged vacuum-driven mode also exhibited rejection over 84% for other smaller polyfluoroalkyl substances (Supplementary Fig. 31).

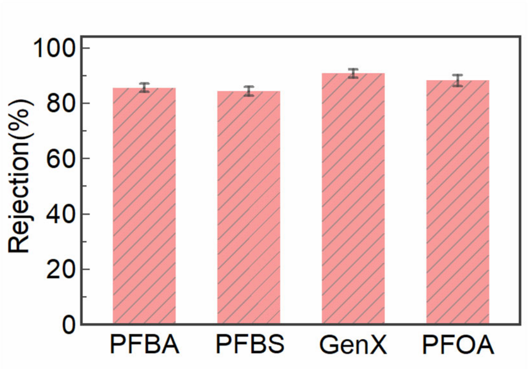

Supplementary Fig. 31 | The PFASs rejection for SNF20-NF0.1* membranes. The error bars of PFASs rejection represent the standard deviation of test date from three distinct samples (n = 3). The rejection tests were performed by a feed solution containing a compound cocktail (200 ppb for each PFASs) mixed with 10 mmol NaCl solution in a submerged vacuum-driven filtration mode with applied vacuum pressure of 0.9 bar.

The digital photo shown in Supplementary Fig. 32 demonstrates a vacuum filtration apparatus comprising a membrane cell immersed in a feed solution, a peristaltic pump providing suction to enable the vacuum-driven nanofiltration process, and an air valve connected to the vacuum system to regulate vacuum pressure. Supplementary Fig. 33 shows the details of the membrane cell, containing a polyacrylic frame (connected to the suction peristaltic pump) and two stainless-steel splints. The NF membranes are sealed between the polyacrylic frame and stainless-steel splints using silicone rubber rings.

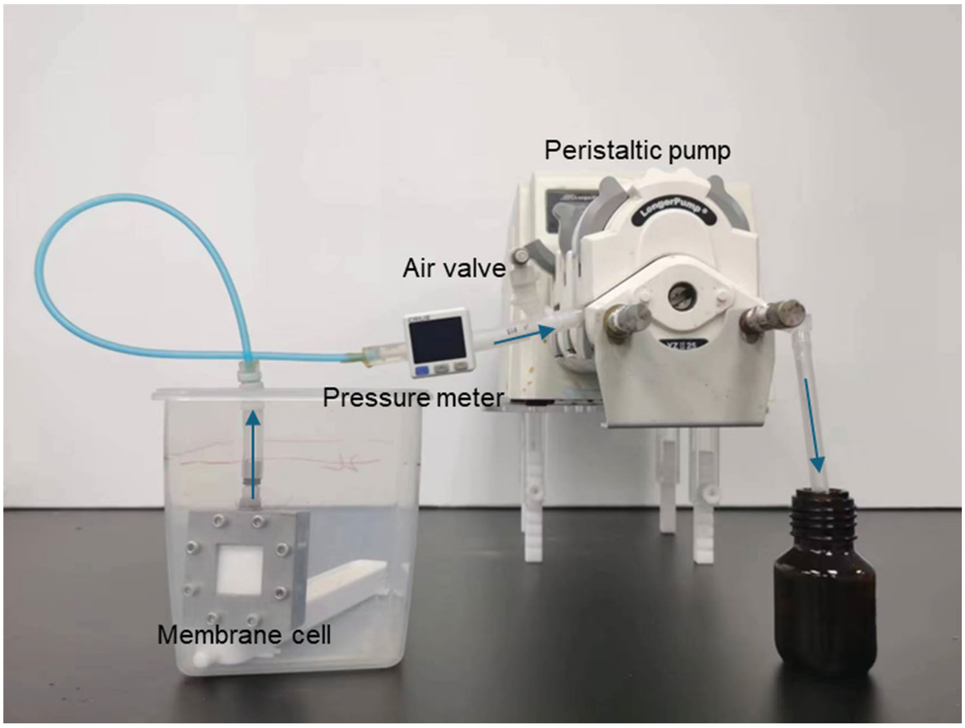

Supplementary Fig. 32 | Digital photo of a vacuum filtration apparatus.

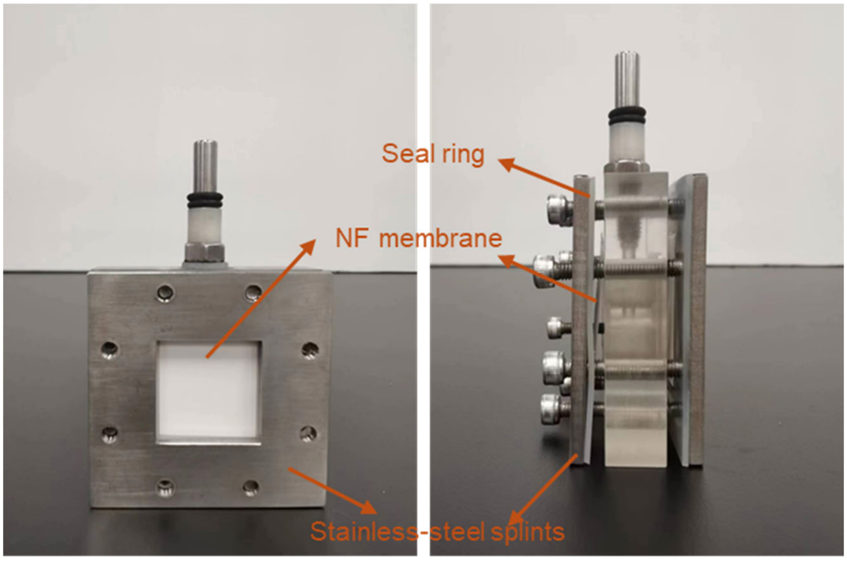

Supplementary Fig. 33 | Digital photo of membrane cell.

The separation performance of SNF20-NF0.1* and NF270 for five salts have been tested in conventional cross-flow filtration mode (Supplementary Fig. 34 and 35). The SNF20-NF0.1* still maintained a Na_2SO_4 rejection of > 96% and a high passage of essential minerals Ca^{2+} and Mg^{2+}, resulting in improved minerals-sulfate selectivity compared to NF270.

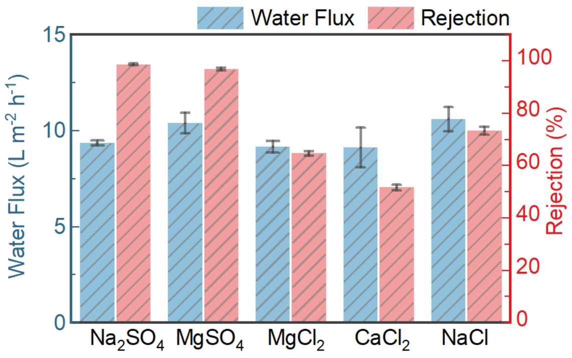

Supplementary Fig. 34 | Separation performance of NF270 membranes for five different salts under the cross-flow filtration mode. The error bars represent the standard deviation of the salt rejection rate from the measurement data of three distinct samples (n = 3, testing condition: single salt concentration of 1000 ppm at a hydraulic pressure of 3 bar).

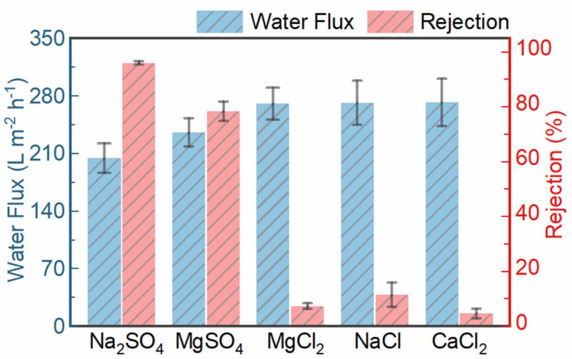

Supplementary Fig. 35 | Separation performance of SNF20-NF0.1* membranes for five different salts under the cross-flow filtration mode. The error bars represent the standard deviation of the salt rejection rate from the measurement data of three distinct samples (n = 3, testing condition: single salt concentration of 1000 ppm at a hydraulic pressure of 3 bar).

We investigated the vacuum filtration performance of SNF20-NF0.1* membrane for a feed solution containing 1000 ppm Na_2SO_4 (Supplementary Fig. 36). The resulting Na_2SO_4 at 0.9 bar of 93.0 ± 0.3% was much lower compared to that of 96.0 ± 0.6% in the cross-flow filtration at 3 bar for the same feed solution. This difference could be attributed to the dilution effect: with a similar solute flux, a lower water flux results in greater permeate concentration and thus reduced salt rejection[3]. In addition, the salt rejection for vacuum filtration of 1000 Na_2SO_4 was also lower than that of 500 Na_2SO_4 (96.3 ± 0.3%).

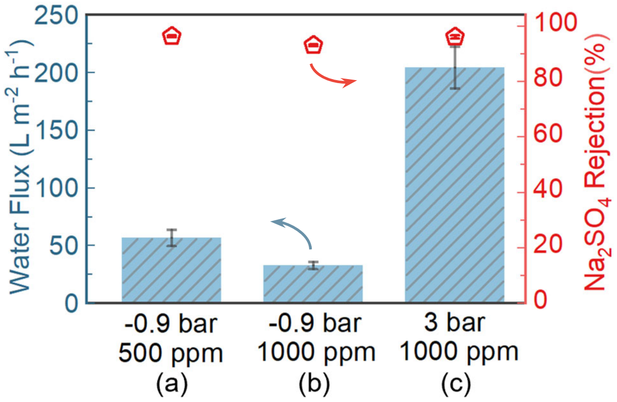

Supplementary Fig. 36 | The separation performance of SNF20-NF0.1* under different operating conditions: (a) vacuum filtration at 0.9 bar for 500 ppm Na2SO4; (b) vacuum filtration at 0.9 bar for 1000 ppm Na_2SO_4; and (c) cross-flow filtration at 3 bar for 1000 ppm Na_2SO_4. The error bars of separation performance represent the standard deviation of the test date from three distinct samples (*n* = 3).

We investigated the influence of operational pressure on the separation performance in two filtration modes. Supplementary Figure 37A demonstrated that increasing the applied vacuum pressure can enhance the apparent Na2SO4 rejection in the vacuum-driven, which can be attributed to the dilution effect[3]. However, in cross-flow mode, the overly high operating pressure (such as 5 bar) may lead to severe concentration polarization that could jeopardize Na_2SO_4 rejection (Supplementary Figure 37B). The SNF20-NF0.1* demonstrated the best Na_2SO_4 rejection at the hydraulic pressure of 3 bar.

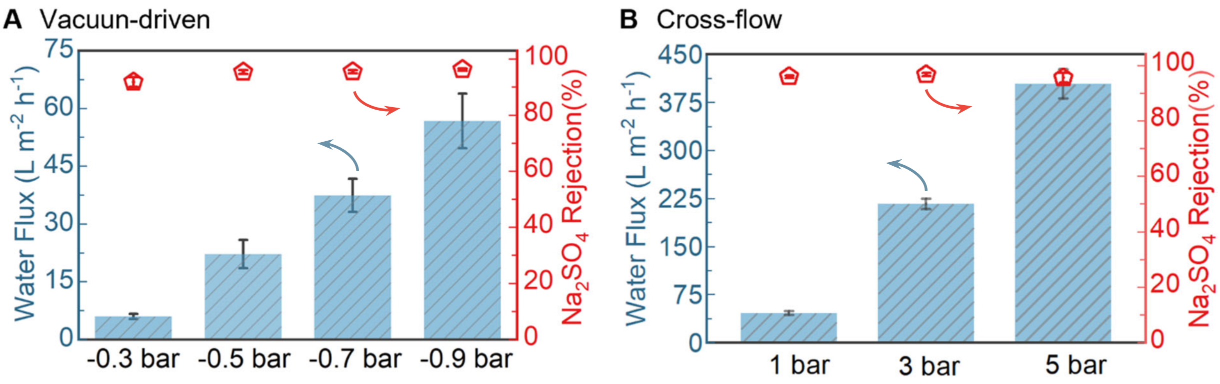

Supplementary Fig. 37 | The influence of operational pressure on the membrane separation performance of SNF20-NF0.1* in two processes. (**A**) Separation performance of SNF20-NF0.1* with different vacuum pressures under the vacuum-driven mode (feed solution of 500 ppm Na_2SO_4). (**B**) Separation performance of SNF20-NF0.1* with different hydraulic pressures under the cross-flow mode (feed solution of 1000 ppm Na_2SO_4). The error bars represent the standard deviation of the salt rejection rate from the measurement data of three distinct samples.

We investigated the influence of Na_2SO_4 concentrations (500 ppm and 1000 ppm) in the two filtration modes. Supplementary Figure 38 demonstrated that increasing feed concentrations decreased the Na_2SO_4 rejections in both cross-flow and vacuum-driven filtration modes.

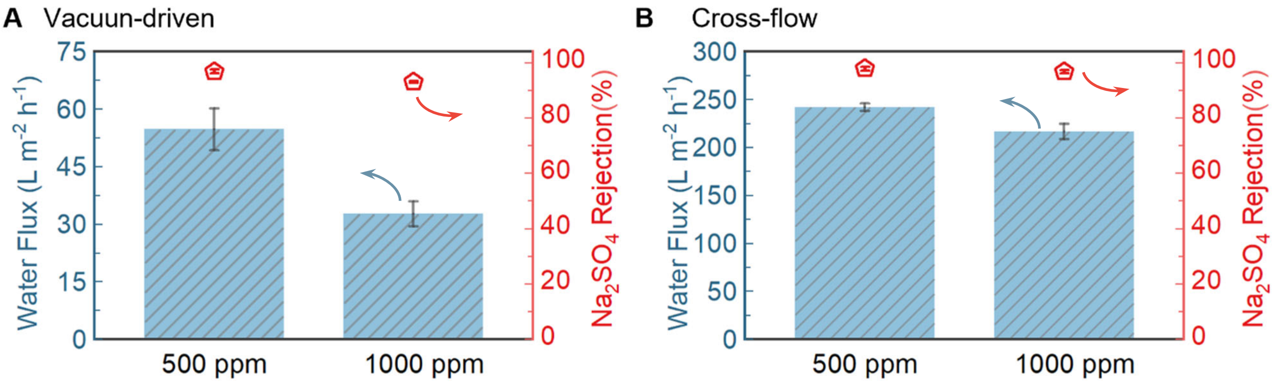

Supplementary Fig. 38 | The influence of the feed concentration of Na_2SO_4 on the membrane separation performance of SNF20-NF0.1*. (**A**) Separation performance of SNF20-NF0.1* with different Na_2SO_4 concentrations under the vacuum-driven mode (hydraulic pressure of -0.9 bar). (**B**) Separation performance of SNF20-NF0.1* with different Na_2SO_4 concentrations in the cross-flow mode (hydraulic pressure of 3 bar). The error bars represent the standard deviation of the salt rejection rate from the measurement data of three distinct samples.

To further confirm the stability of the SNF20-NF0.1*, we tested its separation performance under cross-flow condition over a period of 7 days using a feed solution of 1000 ppm Na_2SO_4 at a hydraulic pressure of 3 bar (Supplementary Fig. 39). The membrane maintained a stable Na_2SO_4 rejection of > 96%. The water flux was slightly reduced, which is likely due to membrane compaction1, 2. The stable Na_2SO_4 rejection implies a good stability of the membrane.

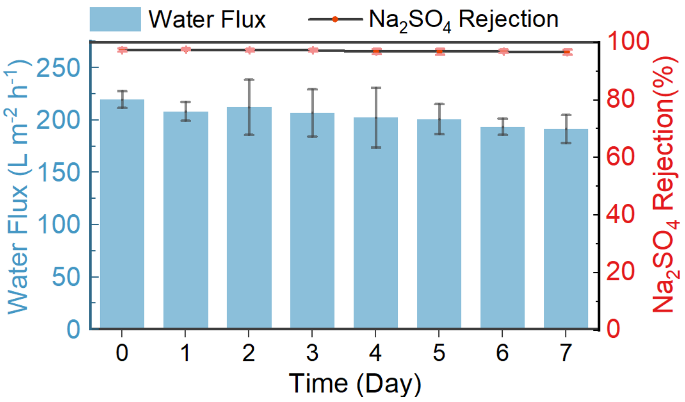

Supplementary Fig. 39 | The long-term separation performance of SNF20-NF0.1*. The rejection test was performed using a feed solution of 1000 ppm Na_2SO_4. The filtration tests adopted a pressure-driven cross-flow mode with an applied hydraulic pressure of 3 bar. The error bars of separation performance represent the standard deviation of data from three distinct samples (n = 3).

3. Supplementary Tables

Supplementary Table 1. Elemental compositions of the top surface of PVDF, SNF-PVDF and SNF0-NF0.5 measured by XPS.

The XPS results	Atom percent (%)				
	O1*s*	N1*s*	C1*s*	F1*s*	O/N
Pristine PVDF	15.92	N.D.[a]	59.05	25.03	N.A.[b]
SNF20-PVDF	17.14	7.69	60.60	14.57	2.23
SNF30-PVDF	17.36	6.54	60.72	15.38	2.66
SNF50-PVDF	20.06	14.54	62.64	2.76	1.38
SNF0-NF0.5 (control)	11.27	4.61	63.38	20.74	2.44

Notes: a. Not detected. b. Not applicable.

Supplementary Table 2. The fabrication recipe for substrates with and without SNF.

Substrates	SNF suspension concentration (ug mL^{-1})	Loading volume (mL)	Loading area (cm^2)	Loading mass per area (ug cm^{-2})
PVDF	129	0	63.5	0
SNF20-PVDF	129	20	63.5	41
SNF30-PVDF	129	30	63.5	61
SNF50-PVDF	129	50	63.5	102

Supplementary Table 3. The fabrication recipe for SNF-NF membranes with and without SNF.

NF membranes	Substrates	PIP concentration (wt%)	TMC concentration (wt%)	IP time (s)
SNF0-NF0.5	PVDF	0.5	0.1	60
SNF20-NF0.5	SNF20-PVDF	0.5	0.1	60
SNF30-NF0.5	SNF30-PVDF	0.5	0.1	60
SNF50-NF0.5	SNF50-PVDF	0.5	0.1	60
SNF0-NF0.1*	PVDF	0.1	0.1	30
SNF0-NF0.2*	PVDF	0.2	0.1	30
SNF20-NF0.1*	SNF20-PVDF	0.1	0.1	30
SNF20-NF0.2*	SNF20-PVDF	0.2	0.1	30

Supplementary Table 4. Operating parameters assumed in the calculation of SEC.

Parameters	*Value*	
Mass transfer coefficient	k	100 L m^{-2} h^{-1}
Transmembrane osmotic pressure difference	π_f	0.1 bar
Water recovery	Y	0.6
Average water flux	J_{av}	25 L m^{-2} h^{-1}

Doppler Broadening Energy Spectroscopy (DBES) is a frequently used technique for characterizing the sub-nanometer pore structure of PA membranes, which is in turn related to their crosslinking degree or density[4]. DBES results (Supplementary Fig. 16) show that the S parameter for both SNF0-NF0.5 and SNF20-NF0.5 membranes ranged from 0.48 to 0.5 within 1 Kev positron energy (which typically detects the signal from the PA rejection layer[4]). The range of S parameters in our study is comparable to those reported in the literature for PA TFC NF membranes (Supplementary Table 5).

Supplementary Table 5. A comparison of S parameters of DBES between this study and other nanofiltration studies in the literature.

Membrane chemistry	Ranges of S parament	References (DOI)
Polyamide	0.48 ~ 0.50	This work
Polyamide	0.48 ~ 0.50	10.1038/s41467-020-15771-2[4]
Polyamide	0.49 ~ 0.51	1 10.1038/s41467-022-28183-1[5]
Polyamide	0.49 ~ 0.51	10.1038/s41467-023-36848-8[6]
Polyamide	0.47 ~ 0.49	10.1038/s41467-023-43291-2[7]
Polyamide	0.47 ~ 0.49	10.3390/polym12102326[8]

Supplementary Table 6 | A comparison of MWCO between this study and other polyamide TFC NF membranes reported in the literature.

Membrane chemistry	MWCO (Da)	References (DOI)
Polyamide	633	This work
Polyamide	600 ~ 800	10.3390/w11122512[9]
Polyamide	500	10.3390/membranes14020038[10]
Polyamide	560	10.1016/j.memsci.2023.122351[11]
Polyamide	725.1	10.1016/j.desal.2023.116575[12]
Polyamide	579	10.1016/j.memsci.2022.121321[13]
Polyamide	534	10.1016/j.memsci.2022.121321[13]
Polyamide	858.9	10.1016/j.memsci.2021.119942[14]
Polyamide	640.6	10.1016/j.memsci.2021.119942[14]
Polyamide	514.2	10.1016/j.memsci.2021.119942[14]
Polyamide	850	10.1016/j.desal.2015.04.027[15]
Polyamide	800	10.1016/j.memsci.2020.117997[16]
Polyamide	639	10.1016/j.seppur.2020.118042[17]
Polyamide	551	10.1016/j.compositesb.2021.108686[18]
Polyamide	726.7	10.1016/j.memsci.2024.122484[19]

NF membranes may have higher Na_2SO_4 rejection compared to that of $MgSO_4$, often due to the Donnan exclusion effect[20, 21]. Supplementary Table 7 listed some PA-based NF membranes with a higher rejection rate for Na_2SO_4 than $MgSO_4$, which are consistent with our rejection results in this work.

Supplementary Table 7. The Na2SO4 and MgSO4 rejection of polyamide NF membranes

Membrane chemistry	Test Conditions (feed concentration; applied pressure)*	Na_2SO_4 Rejection (%)	$MgSO_4$ Rejection (%)	References (DOI)
Polyamide	1000 ppm; 5 bar	99.6	93.2	10.1126/science.adi9531[22]
Polyamide	2000 ppm; 10 bar	96.0	93.4	10.1038/s41467-020-19809-3[23]
Polyamide	1000 ppm, 4 bar	95.3	~90.0	10.1038/s41467-018-04467-3[24]
Polyamide	2000 ppm; 5 bar	94.0	84.8	10.1039/d1ta04763a[25]
Polyamide	2000 ppm; 5 bar	98.8	85.5	10.1039/d1ta04763a[25]
Polyamide	1000 ppm; 5 bar	95.5	~80.0	10.1016/j.memsci.2020.117971[26]
Polyamide	1500 ppm ; 4 bar	97.5	91.2	10.1039/c9ta02299f[27]
Polyamide	1000 ppm ; 2 bar	92.0	67.6	10.1016/j.memsci.2017.09.016[28]
Polyamide	1000 ppm; 6 bar	99.7	86.0	10.1039/c7ta00501f[29]
Polyamide	1000 ppm; 5 bar	94.8	72.5	10.1007/s10853-018-2369-2[30]
Polyamide	1000 ppm; 2 bar	87.6	~71.0	10.1016/j.memsci.2013.12.060[31]

* The concentrations are the salt concentrations in a single salt separation test; the pressures are the applied hydraulic pressure under cross-flow filtration mode. In addition, all

membranes listed in the table are PA TFC NF membranes with a negative surface zeta potential at the tested condition.

4. Supplementary References

1. Xin W, *et al.* High-performance silk-based hybrid membranes employed for osmotic energy conversion. *Nature Communications* **10**, (2019).

2. Abdullah SZ, Bérubé PR, Horne DJ. SEM imaging of membranes: Importance of sample preparation and imaging parameters. *Journal of Membrane Science* **463**, 113-125 (2014).

3. Yang Z, Guo H, Tang CY. The upper bound of thin-film composite (TFC) polyamide membranes for desalination. *Journal of Membrane Science* **590**, 117297 (2019).

4. Liang Y, *et al.* Polyamide nanofiltration membrane with highly uniform sub-nanometre pores for sub-1 Å precision separation. *Nature Communications* **11**, (2020).

5. Shen L, *et al.* Polyamide-based membranes with structural homogeneity for ultrafast molecular sieving. *Nature Communications* **13**, 500 (2022).

6. Zhao C, *et al.* Polyamide membranes with nanoscale ordered structures for fast permeation and highly selective ion-ion separation. *Nature Communications* **14**, 1112 (2023).

7. Zhao G, Gao H, Qu Z, Fan H, Meng H. Anhydrous interfacial polymerization of sub-1 Å sieving polyamide membrane. *Nature Communications* **14**, 7624 (2023).

8. Ang MBMY, *et al.* Surface Properties, Free Volume, and Performance for Thin-Film Composite Pervaporation Membranes Fabricated through Interfacial Polymerization Involving Different Organic Solvents. *Polymers* **12**, 2326 (2020).

9. Cooray T, *et al.* Drinking-Water Supply for CKDu Affected Areas of Sri Lanka, Using Nanofiltration Membrane Technology: From Laboratory to Practice. *Water* **11**, 2512 (2019).

10. Chidichimo F, Basile MR, Conidi C, De Filpo G, Morelli R, Cassano A. A New Approach for Bioremediation of Olive Mill Wastewaters: Combination of Straw Filtration and Nanofiltration. *Membranes (Basel)* **14**, (2024).

11. Zhang Y, *et al.* Based on high cross-linked structure design to fabricate PEI-based nanofiltration membranes for Mg2+/Li+ separation. *Journal of Membrane Science* **693**, 122351 (2024).

12. Li T, Zhang X, Zhang Y, Wang J, Wang Z, Zhao S. Nanofiltration membrane comprising

structural regulator Cyclen for efficient Li+/Mg2+ separation. *Desalination* **556**, 116575 (2023).

13. Li H, Li Y, Li M, Jin Y, Kang G, Cao Y. Improving Mg2+/Li+ separation performance of polyamide nanofiltration membrane by swelling-embedding-shrinking strategy. *Journal of Membrane Science* **669**, 121321 (2023).

14. Wu M-B*, et al.* Positively-charged nanofiltration membranes constructed via gas/liquid interfacial polymerization for Mg2+/Li+ separation. *Journal of Membrane Science* **644**, 119942 (2022).

15. Li X, Zhang C, Zhang S, Li J, He B, Cui Z. Preparation and characterization of positively charged polyamide composite nanofiltration hollow fiber membrane for lithium and magnesium separation. *Desalination* **369**, 26-36 (2015).

16. Wu H*, et al.* A novel nanofiltration membrane with [MimAP][Tf2N] ionic liquid for utilization of lithium from brines with high Mg2+/Li+ ratio. *Journal of Membrane Science* **603**, 117997 (2020).

17. Xu P, Hong J, Xu Z, Xia H, Ni Q-Q. Novel aminated graphene quantum dots (GQDs-NH2)-engineered nanofiltration membrane with high Mg2+/Li+ separation efficiency. *Separation and Purification Technology* **258**, 118042 (2021).

18. Xu P, Hong J, Xu Z, Xia H, Ni Q-Q. MWCNTs-COOK-assisted high positively charged composite membrane: Accelerating Li+ enrichment and Mg2+ removal. *Composites Part B: Engineering* **212**, 108686 (2021).

19. Zha Z, Li T, Hussein I, Wang Y, Zhao S. Aza-crown ether-coupled polyamide nanofiltration membrane for efficient Li+/Mg2+ separation. *Journal of Membrane Science* **695**, 122484 (2024).

20. Schaep J, Van der Bruggen B, Vandecasteele C, Wilms D. Influence of ion size and charge in nanofiltration. *Separation and Purification Technology* **14**, 155-162 (1998).

21. Wang J*, et al.* Graphene Oxide as an Effective Barrier on a Porous Nanofibrous Membrane for Water Treatment. *ACS Applied Materials & Interfaces* **8**, 6211-6218 (2016).

22. Zhang Y*, et al.* Ice-confined synthesis of highly ionized 3D-quasilayered polyamide nanofiltration membranes. *Science* **382**, 202-206 (2023).

23. Yuan B, Zhao S, Hu P, Cui J, Niu QJ. Asymmetric polyamide nanofilms with highly

ordered nanovoids for water purification. *Nature Communications* **11**, 6102 (2020).

24. Wang Z*, et al.* Nanoparticle-templated nanofiltration membranes for ultrahigh performance desalination. *Nature Communications* **9**, 2004 (2018).

25. Sarkar P, Modak S, Ray S, Adupa V, Reddy KA, Karan S. Fast water transport through sub-5 nm polyamide nanofilms: the new upper-bound of the permeance–selectivity trade-off in nanofiltration. *J Mater Chem A* **9**, 20714-20724 (2021).

26. Yang S, Wang J, Fang L, Lin H, Liu F, Tang CY. Electrosprayed polyamide nanofiltration membrane with intercalated structure for controllable structure manipulation and enhanced separation performance. *Journal of Membrane Science* **602**, 117971 (2020).

27. Zhu J*, et al.* MOF-positioned polyamide membranes with a fishnet-like structure for elevated nanofiltration performance. *J Mater Chem A* **7**, 16313-16322 (2019).

28. Wu M*, et al.* Fabrication of composite nanofiltration membrane by incorporating attapulgite nanorods during interfacial polymerization for high water flux and antifouling property. *Journal of Membrane Science* **544**, 79-87 (2017).

29. Wang J-J, Yang H-C, Wu M-B, Zhang X, Xu Z-K. Nanofiltration membranes with cellulose nanocrystals as an interlayer for unprecedented performance. *J Mater Chem A* **5**, 16289-16295 (2017).

30. Wei C*, et al.* One-step fabrication of recyclable polyimide nanofiltration membranes with high selectivity and performance stability by a phase inversion-based process. *Journal of Materials Science* **53**, 11104-11115 (2018).

31. Li Y*, et al.* Surface fluorination of polyamide nanofiltration membrane for enhanced antifouling property. *Journal of Membrane Science* **455**, 15-23 (2014).
